# Comparison of alveolar bone width and sagittal tooth angulation of maxillary central incisors in Class I and Class III canine relationships: a retrospective study using CBCT

**DOI:** 10.1186/s12903-022-02331-x

**Published:** 2022-07-22

**Authors:** Chen Lei, Qun Yu, Di Wu, Kunzhan Cai, Paul Weigl, Chunbo Tang

**Affiliations:** 1 Department of Dental Implantology, The Affiliated Stomatological Hospital of Nanjing Medical University, Jiangsu Province Key Laboratory of Oral Diseases, Jiangsu Province Engineering Research Center of Stomatological Translational Medicine, Nanjing, 210029 China; 2 Faculty of Oral and Dental Medicine, J.W. Goethe University, Frankfurt am Main, Germany

**Keywords:** Canine relationship, Cone-beam computed tomography, Immediate implant, Maxillary central incisor

## Abstract

**Background:**

Canine relationship is a key reference identifying anterior malocclusion and an important implication for evaluating preimplantation bone morphology at maxillary esthetic zone. This study aimed to compare the differences of maxillary central incisor-related measurements (alveolar bone thickness and tooth sagittal angulation) between Class I and Class III canine relationship and further explore the risk factors for immediate implant placement in the anterior maxilla based on cone beam computed tomography (CBCT) data.

**Methods:**

CBCT digital imaging and communications in medicine (DICOM) files of 107 patients (54 with Class I canine relationship and 53 with Class III canine relationship) were collected and the alveolar bone thickness at mid-root (mid-root buccal thickness/MBT; palatal/MPT), apical regions (apical buccal thickness/ABT; palatal/APT) and sagittal angulation (SA) of the maxillary central incisor at the examined side were measured on the mid-sagittal observation plane. Descriptive statistical analysis and frequency distributions of the measurements based on Class I or Class III canine relationship were established. Statistical analyses were performed using Fisher’s exact test, independent samples t test and Pearson correlation test with the significance level set at *p* < 0.05.

**Results:**

The frequency distributions of maxillary central incisors’ MPT, ABT, APT and SA showed significant differences between Class I and Class III canine relationships (*p* = 0.030, 0.024, 0.000 and 0.000, respectively). MPT (2.48 ± 0.88 mm vs. 3.01 ± 1.04 mm, *p* = 0.005), APT (6.79 ± 1.65 mm vs. 8.47 ± 1.93 mm, *p* = 0.000) and SA (12.23 ± 5.62° vs. 16.42 ± 4.49°, *p* = 0.000) were significantly smaller in patients with Class III canine relationship. Moreover, SA showed a strong positive correlation with APT (R = 0.723, *p* = 0.000) and a moderate negative correlation with ABT (R = − 0.554, *p* = 0.000).

**Conclusions:**

In populations with Class III canine relationship, maxillary central incisors were significantly more labially inclined and have a thinner palatal bone plate at the apex compared with Class I relationship. Clinicians should avoid palatal perforation during immediate implantation at sites of originally protrusive maxillary incisors.

## Background

Clinical outcomes of conventional implant restoration may be impeded by compromised bone volume due to horizontal and vertical dimension loss after tooth extraction. In 1978, Schulte for the first time achieved successful implant placement into fresh extraction sockets and introduced the concept “immediate implant” [1] which could achieve favorable peri-implant bony response, soft tissue levels and overall aesthetics in the mid-long term [2]. However, there were still some limitations for immediate implantation. According to the 5th ITI Consensus Conference in 2013, immediate implant placement required a labial bone wall at least 1 mm thick [3]. Studies have shown that 53% of maxillary central incisors presented facial bone defects at extraction, and 85.3% had a buccal bone wall less than 1 mm at crest level [4]. Therefore, sufficient palatal and apical bone volumes of extraction socket appeared to be important for implant engagement [3, 5–7].

Alveolar bone thickness can be influenced by maxillofacial characteristics such as sagittal facial pattern and vertical growth pattern [8]. Hyperdivergent individuals were reported to have thinner alveolar bone than hypodivergent individuals [8–10]. According to Chung et al., the alveolar bone height and thickness of patients with skeletal class III high-angle occlusion were significantly smaller than those with skeletal class III average-angle and normal occlusion [11]. Apart from skeletal patterns, factors like dentoalveolar compensations in different types of malocclusion can also affect alveolar bone morphology. Evangelista et al. found Class I malocclusion patients had a 35% higher prevalence of alveolar dehiscence than those with Class II division 1 malocclusion [12]. In the anterior maxilla, frontal teeth function as an integrated part in guiding mandibular movements, among which, canines serve as an important reference in Angle’s classification of the anterior malocclusion. Incorrect canine relationships are commonly encountered along with retroclined or proclined incisors, which were reported to significantly influence the surrounding alveolar bone morphology and thickness [13–15].

Based on the facts above, it is reasonable to assume that the maxillary anterior teeth’s bone volume differs between different canine relationships. Canine relationship can be considered as an important predicting factor for alveolar bone volume at anterior maxilla and serve as an indicator in helping surgeons with immediate implant patient selection and treatment planning.

To the best of our knowledge, few studies have compared the differences in alveolar bone morphology and tooth angulation of maxillary anterior teeth in different canine relationships. Since distal canine (Class II) relationship can be seen in both Angle Class II, division 1 and 2 malocclusions, in which upper incisors can be either labially or palatally inclined influencing tooth angulation. Therefore, in this study, only cone-beam computed tomography (CBCT) data of patients with mesial (Class III) and neutral (Class I) canine relationships were collected and the alveolar bone wall thickness of maxillary central incisors and the tooth inclination within their bony housing were measured based on CBCT images. This study aimed to investigate the correlation between these parameters and canine relationships to further assess the related factors affecting the bone volume of maxillary central incisors and explore risk factors for immediate implant placement in the anterior esthetic zone.

## Methods

### Patients

This retrospective study was approved by the Ethics Committee of the Affiliated Hospital of Stomatology, Nanjing Medical University (Approval number PJ2019-092-001). A total of 107 CBCT images of Chinese patients (54 men and 53 women) were selected from the Department of Oral and Maxillofacial Radiology, Affiliated Hospital of Stomatology, Nanjing Medical University, from June 2019 to June 2021. The CBCT images were taken for orthodontic or implantation purposes. The selected subjects met the following inclusion criteria: (a) permanent maxillary and mandibular incisors and canines are present bilaterally. (b) canine relationships of both sides are the same and classified as neutral or mesial; (c) age ≥ 18 years at the time of evaluation; (d) no history of systemic disease, orthodontic treatment, trauma, or periodontal surgery in the region of interest; (e) bone tissue around the examined teeth should be intact and show no evidence of space-occupying lesions, apical lesions, surgical treatment, dental trauma or bone resorption. Scattered, distorted or blurred images were excluded. Teeth with severe displacement or rotation were also excluded. According to previous studies, there were no significant differences between the measurement values on the right and left sides [16, 17]; therefore, in the current study, only the side of each subject’s CBCT images with a clearer canine relationship and less crowded teeth was chosen for analysis [18].

### CBCT process

All images were acquired with a CBCT machine (Newtom VGi evo; NewTom Inc, Italy). Imaging parameters were set at 110 kV, 5 mA, scan time 20 s, resolution 0.3 mm. Patients’ head positions were standardized with respect to the horizontal and vertical reference lines before the CBCT images were taken. All the data were transmitted in digital imaging and communication in medicine (DICOM) format, and then reconstructed and analyzed using a 3D-segmentation and dental planning software (SIMPLANT Pro, version 17.01; Materialise, Belgium). CBCT data were first reconstructed with 0.3-mm-thick slices, then 3D reconstruction was established by setting the threshold value above 1000 to show the clear alignment of teeth using the “Segment” tool in SIMPLANT software.

### Canine relationship determination

**Fig. 1:**
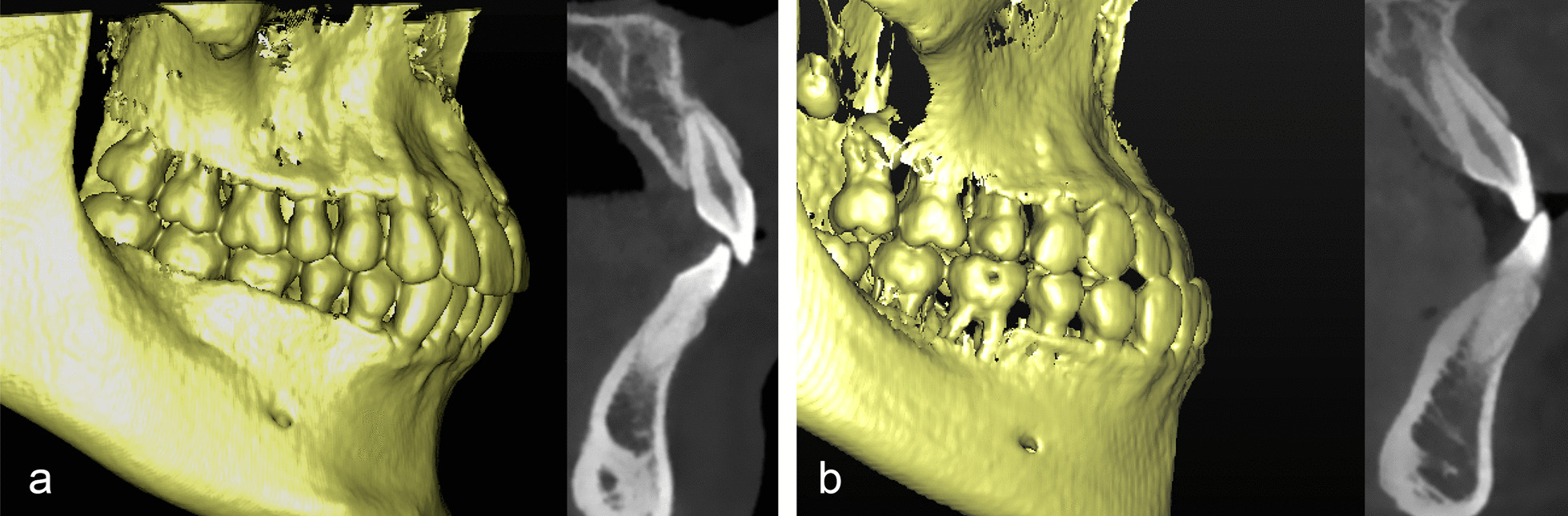
**a** Example of neutral canine relationship (Class I) and corresponding maxillary central incisor; **b** Example of mesial canine relationship (Class III) and corresponding maxillary central incisor

Next, the canine relationships of all patients were determined by a single dental clinician. The assessment criteria were as follows: (1) Neutral relationship (Class I): with the mandible in centric relation position, the cusp tip of the upper canine is in contact with the distal surface of the lower canine, and the mesial incline of the upper canine occludes with the distal incline of the lower canine (Fig. 1a). (2) Mesial relationship (Class III): with the mandible in centric relation position, the cusp tip of the upper canine is distal to the lower canine and is in contact with the mandibular first premolar, and anterior teeth are in crossbite (Fig. 1b). The patients were classified into two groups on the basis of canine relationship. The Class I canine relationship group comprised 54 patients (27 males and 27 females); the Class III canine relationship group comprised 53 patients (27 males and 26 females).

## CBCT measurements

**Fig. 2:**
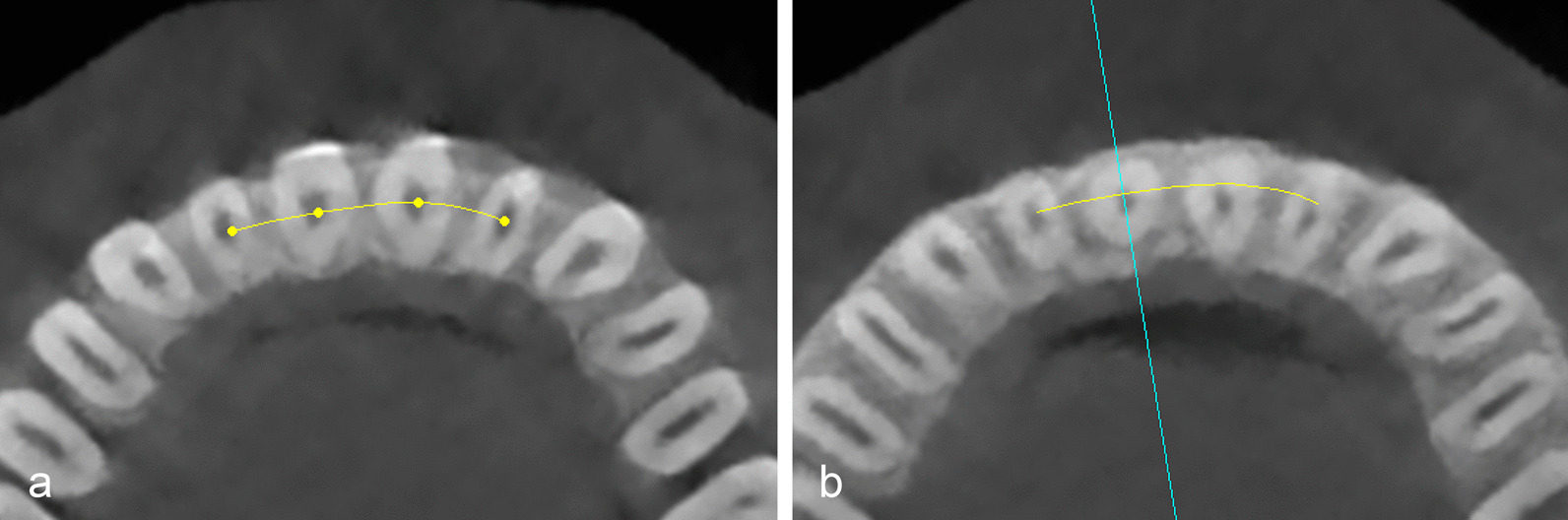
**a** Determination of panoramic curve after head position reorientation; **b** Observation plane (blue): mid-sagittal plane passes through chosen tooth’s long axis which was perpendicular to panoramic curve (yellow)

Before CBCT measurements, reorientation of slice images to the natural head position was performed by rotating coronal and cross-sectional slices, after which a panoramic curve was drawn connecting the occluding center of each maxillary incisor (#12–22) (Fig. 2a). The mid-sagittal plane passing through the long axis of the examined maxillary central incisor was defined as the observation plane, which was perpendicular to the panoramic curve (Fig. 2b).

**Fig. 3:**
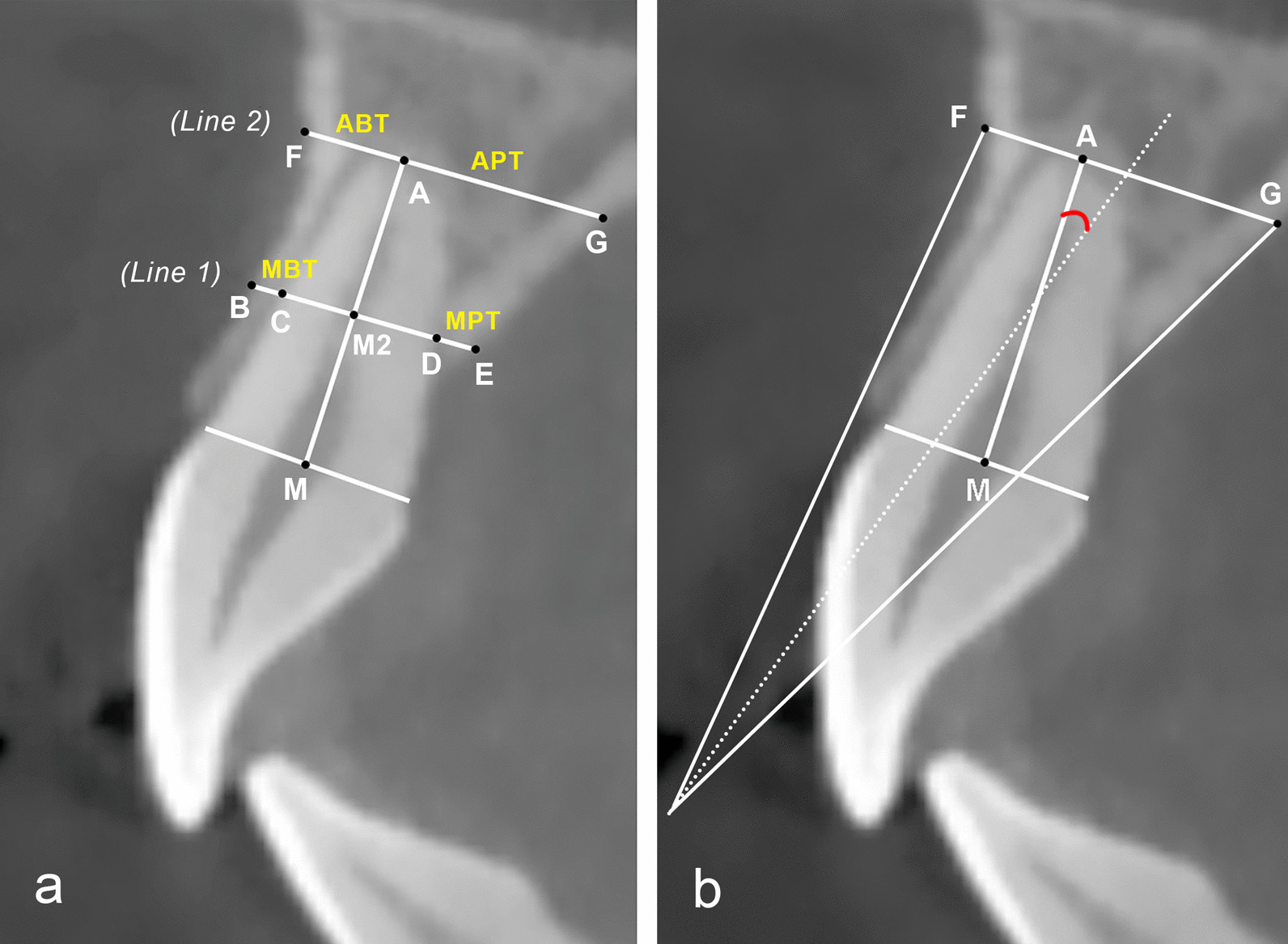
**a** Measurements of bone thickness at different aspects; **b** Measuring the sagittal angulation of the maxillary central incisor based on the tooth axis and the axis of its respective alveolar process. MBT, mid-root buccal thickness; MPT, mid-root palatal thickness; ABT, apical buccal thickness; APT, apical palatal thickness

The landmarks were identified and marked as previously described (Fig. 3a) [19]. Point M: the midpoint of the line joining the buccal and palatal cementoenamel junction of the tooth; Point A: the apical point of the tooth; according to a previous study, only a mean angle of 1.74° is present between the long axis of the root and the crown of normal maxillary central incisors [20]. Therefore, in the present study, Line AM was identified as the long axis of the tooth rather than the axis of the root. Line 1 was made perpendicular to the tooth axis passing through the midpoint (point M2) of Line AM. Points B, C, D and E represent the intersections between Line 1 and the outer surface of the buccal bone, the buccal root surface, the palatal root surface and the outer surface of the palatal bone, respectively. The mid-root buccal thickness (MBT) was measured from points B to C and the mid-root palatal thickness (MPT) was measured from points E to D. Line 2 was made perpendicular to the tooth axis passing through point A. Points F and G represent the intersections between Line 2 and the outer surface of the buccal bone plate and the outer surface of the palatal bone plate, respectively. The apical buccal thickness (ABT) was measured from points A to F and the apical palatal thickness (APT) was measured from points A to G. The sagittal angulation (SA) of the tooth was measured as the angle between the long axes of the tooth and its respective alveolar process (Fig. 3b), which was marked by bisecting the buccal line (the line connecting point F and the outer buccal alveolar crest) and the palatal line (the line connecting point G and the outer palatal alveolar crest).

All measurements were conducted by a single dental clinician. To check the intraexaminer reliability, ten randomly selected CBCT scans, and five measurements (MBT, MPT, ABT, APT and SA) were assessed twice at a 4-week interval between them. The intraclass correlation coefficient (ICC) was 0.982 (*p* = 0.000).

### Statistical analysis

Descriptive statistical analysis was performed to show the mean thickness of MBT, MPT, ABT and APT, respectively. The frequency distributions of bone widths and SA of the upper central incisors according to Class I or Class III canine relationship were established and analyzed with Fisher’s exact test. Independent samples t tests were used to compare the bone thickness and angulation between two different canine relationships. The Pearson correlation test was used to confirm the correlation between tooth inclination and thickness. All statistical analyses were implemented with SPSS (IBM SPSS Statistics, version 22.0; IBM Corp, Chicago, Illinois) with the significance level set at *p* < 0.05.

## Results

The Class I canine relationship group comprised 54 patients (27 males and 27 females); the Class III canine relationship group comprised 53 patients (27 males and 26 females). Table 1 shows sample characteristics according to canine relationship and indicates that age did not differ significantly between Class I and Class III canine relationship (*P* > 0.05).

**Table 1:** Sample characteristics

Descriptive values	Class I	Class III	p*
Sex, n (%)	Male: 27 (50%)	Male: 27 (50.9%)	
	Female: 27 (50%)	Female: 26 (49.1%)	
Age, year (mean ± SD)	25.54	23.60	0.06

*Independent samples *t* test

**Fig. 4:**
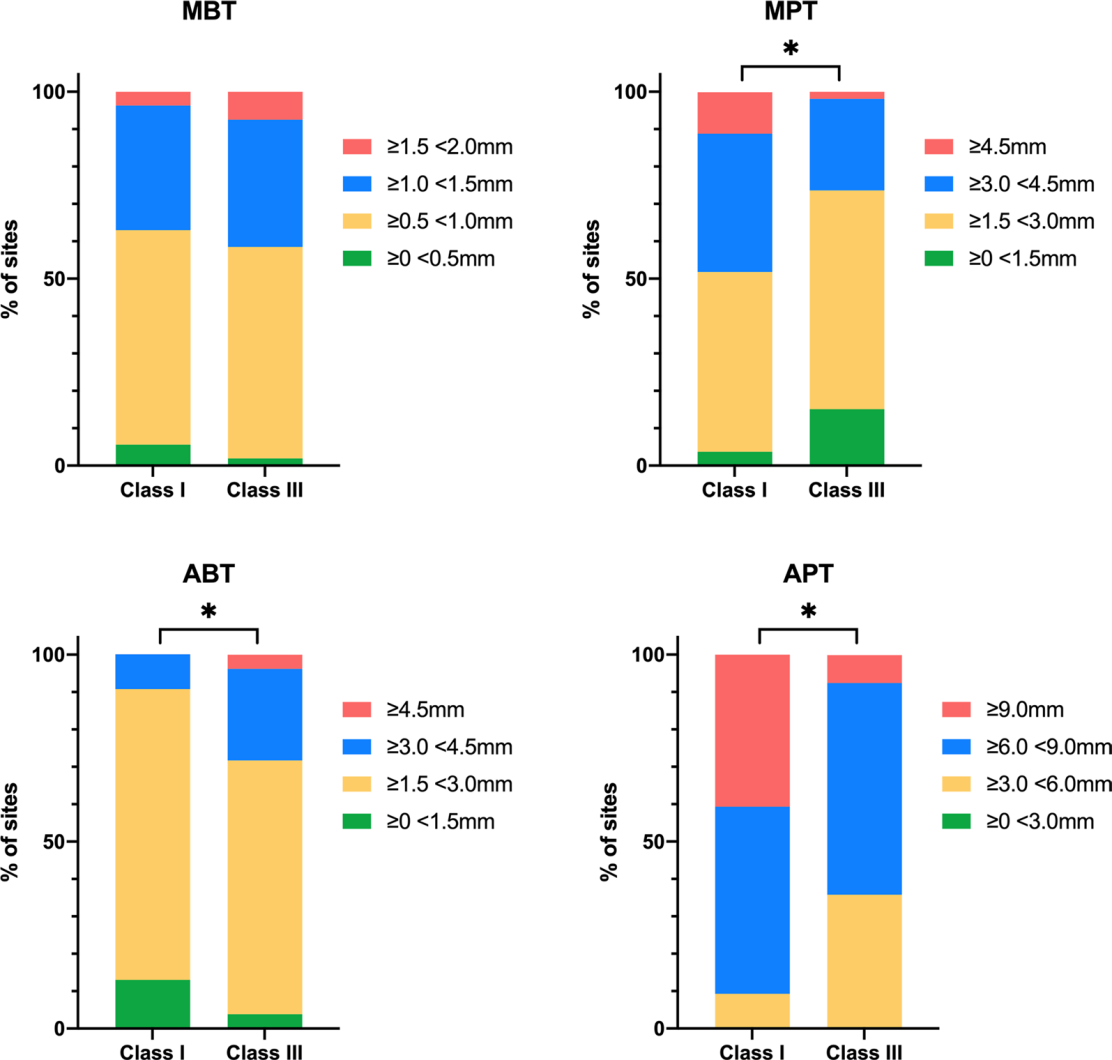
Illustration of the frequency distributions of MBT, MPT, ABT, APT. MBT, mid-root buccal thickness; MPT, mid-root palatal thickness; ABT, apical buccal thickness; APT, apical palatal thickness. *Statistically significant difference between Class I and III canine relationships (*p* < 0.05)

The descriptive statistics results of the mean values of MBT, MPT, ABT and APT regardless of different canine relationships are shown in Table 2. The distributions of the four bone thickness measurements aforementioned in Class I and III canine relationships were established (Fig. 4). As depicted in Table 3, the distributions of MPT (*P* = 0.030), ABT (*p* = 0.024) and APT (*p* = 0.000) showed statistically significant differences between the two different canine relationship populations. Thinner MPT and APT accounted for a larger percentage in Class III group. The most prevalent range of MBT in Class I and Class III were both 0.5–1 mm, accounting for 57.4% and 56.6%, respectively. ABT between 1.5 and 3.0 mm had the highest prevalence in Class I (77.8%) and III (67.9%) populations. In Class I population, 13% of ABT presented less than 1.5 mm, while the incidence for Class III patients accounted for only 3.8%. APT predominantly ranged from 6.0 to 9.0 mm in both Class I (50.0%) and Class III (56.6%) population. The percentage of APT ≥ 9.0 mm in Class III patients was only 7.5%, which was nearly one-fifth of its counterpart in Class I (40.7%).

**Table 2:** Alveolar bone thicknesses around upper central incisors and *p* values from independent samples t test for comparisons between Class I and III canine relationships

Measurements (mm)	Mid-root bone plate width		Apical bone plate width	
	Buccal (MBT)	Palatal (MPT)	Buccal (ABT)	Palatal (APT)
Mean ± SD	0.97 ± 0.32	2.75 ± 1.00	2.37 ± 0.86	7.64 ± 1.98
Median	0.94	2.77	2.23	7.64
Max	1.71	5.45	5.36	14.16
Min	0.00	0.00	0.28	3.72
Class I (mean ± SD)	0.91 ± 0.33	3.01 ± 1.04	2.23 ± 0.70	8.47 ± 1.93
Class III (mean ± SD)	1.02 ± 0.31	2.48 ± 0.88	2.52 ± 0.98	6.79 ± 1.65
*p*	0.085	0.005^*^	0.084	0.000^*^

*MBT* mid-root buccal thickness, *MPT* mid-root palatal thickness, *ABT* apical buccal thickness, *APT* apical palatal thickness, *SD* standard deviation

*Statistically significant (*p* < 0.05)

**Table 3:** Distributions of four alveolar bone thicknesses around upper central incisors and *p* values from Fisher’s exact test for comparisons between Class I and III canine relationships

	Class I	Class III	Total	*p*
MBT				
≥ 0 < 0.5 mm	3 (5.6%)	1 (1.9%)	4 (3.7%)	0.699
≥ 0.5 < 1.0 mm	31 (57.4%)	33 (56.6%)	64 (57.0%)	
≥ 1.0 < 1.5 mm	18 (33.3%)	18 (34.0%)	36 (33.6%)	
≥ 1.5 < 2.0 mm	2 (3.7%)	4 (7.5%)	6 (5.6%)	
Total	54 (100.0%)	53 (100.0%)	107 (100.0%)	
MPT				
≥ 0 < 1.5 mm	2 (3.7%)	8 (15.1%)	10 (9.3%)	0.030*
≥ 1.5 < 3.0 mm	26 (48.1%)	31 (58.5%)	57 (53.3%)	
≥ 3.0 < 4.5 mm	20 (37.0%)	13 (24.5%)	33 (30.8%)	
≥ 4.5 mm	6 (11.1%)	1 (1.9%)	7 (6.5%)	
Total	54 (100.0%)	53 (100.0%)	107 (100.0%)	
ABT				
≥ 0 < 1.5 mm	7 (13.0%)	2 (3.8%)	9 (8.4%)	0.024*
≥ 1.5 < 3.0 mm	42 (77.8%)	36 (67.9%)	78 (72.9%)	
≥ 3.0 < 4.5 mm	5 (9.3%)	13 (24.5%)	18 (16.8%)	
≥ 4.5 mm	0 (0.0%)	2 (3.8%)	2 (1.9%)	
Total	54 (100.0%)	53 (100.0%)	107 (100.0%)	
APT				
≥ 0 < 3.0 mm	0 (0.0%)	0 (0.0%)	0 (0.0%)	0.000*
≥ 3.0 < 6.0 mm	5 (9.3%)	19 (35.8%)	24 (22.4%)	
≥ 6.0 < 9.0 mm	27 (50.0%)	30 (56.6%)	57 (53.3%)	
≥ 9.0 mm	22 (40.7%)	4 (7.5%)	26 (24.3%)	
Total	54 (100.0%)	53 (100.0%)	107 (100.0%)	

*MBT* mid-root buccal thickness, *MPT* mid-root palatal thickness, *ABT* apical buccal thickness, *APT* apical palatal thickness

*Statistically significant (*p* < 0.05)

**Fig. 5:**
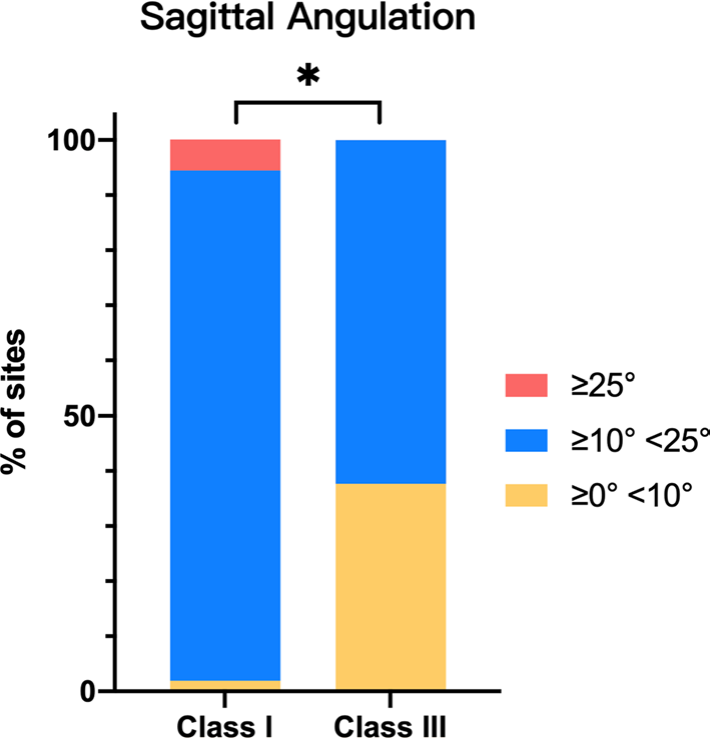
Illustration of the frequency distribution of sagittal angulation between the long axes of tooth and its associated alveolar bone. *Statistically significant difference between Class I and III canine relationships (*p* < 0.05)

As shown in Table 4, the mean SA of the maxillary central incisor with Class I canine relationship was significantly greater than that with Class III relationship (*p* = 0.000). Table 5 and Fig. 5 show that the distribution of maxillary central incisor’s SA was significantly different between Class I and III canine relationship populations (*p* = 0.000). The correlations between bone thicknesses and SA were detected using the Pearson correlation test. Only correlation coefficients R greater than ± 0.5 with a significance level *p* < 0.05 were considered meaningful. Table 6 shows a strong positive correlation between SA and APT (R = 0.723, *p* = 0.000) and a moderate negative correlation between SA and ABT (R = − 0.554, *p* = 0.000).

**Table 4:** Mean ± SD of the SA of upper central incisor and *p* value from independent samples *t* test for comparison between Class I and III canine relationships

	Class I (mean ± SD)	Class III (mean ± SD)	*p*
SA	16.42 ± 4.49°	12.23 ± 5.62°	0.000*

*SA* sagittal angulation, *SD* standard deviation

*Statistically significant (*p* < 0.05)

**Table 5:** Distribution of the SA of upper central incisor and *p* value from Fisher’s exact test for comparison between Class I and III canine relationships

	Class I	Class III	Total	*p*
≥ 0° <10°	1 (1.9%)	20 (37.7%)	21 (19.6%)	0.000*
≥ 10° <25°	50 (92.6%)	33 (62.3%)	83 (77.6%)	
≥ 25°	3 (5.6%)	0 (0.0%)	3 (2.8%)	
Total	54 (100.0%)	53 (100.0%)	107 (100.0%)	

*SA* sagittal angulation

*Statistically significant (*p* < 0.05)

## Discussion

In the present study, we investigated the correlation between canine relationship (Class I or III) and maxillary central incisor’s alveolar bone thicknesses and its sagittal angulation based on CBCT images. Our findings asserted that canine relationship can be considered a predictor for alveolar bone volume in upper incisors and Class III canine relationship is a risk factor for immediate implant placement at anterior maxilla.

All the included patients presented a relatively thin buccal bone plate at the mid-root level (0.97 ± 0.32 mm) irrespective of canine relationship (Table 2). More than half of the population with Class I (63%) or Class III (58.5%) canine relationships could not meet the requirement for immediate implant placement (intact facial bone > 1 mm) in the maxillary esthetic zone at the mid-root level (Table 3), which is the most common site of occurrence of fenestration and perforation [19]. A similar width distribution tendency was observed in a previous radiographic study which showed that 89.3% of central incisors had a thin facial bone wall less than 1 mm at the middle of the root [16]. Identically, another research reported that a thin facial bone wall predominated (≤ 1 mm) at the mid-root level (92%) in maxillary frontal teeth [21]. The proportion differences between the results of previous and current studies may be due to different reference lines, tooth sites of interest and patient ethnic groups.

Angle Class III canines are one of the common dental features of Class III malocclusion, which also include Angle Class III molars, retroclined lower incisors and proclined upper incisors, an edge-to-edge incisor relationship or a negative overjet [22]. Sendyk et al. [23] found that in subjects with Class III dentofacial deformities, maxillary central incisors’ average buccolingual inclination was significantly greater than normal occlusion (*p* < 0.001) and the palatal alveolar thickness at apical (8 mm from the CEJ) was significantly lower (*p* < 0.001). In another study, it was found that increased buccolingual angulation of the maxillary lateral incisors in relation to the palatal plane was correlated with a thinner apical palatal bone plate in an Asian population (Pearson correlation coefficient R = − 0.517 and − 0.579 for males and females) [15]. Similarly, our findings showed that SA of maxillary central incisors with Class III canine relationship were significantly smaller than Class I (12.23 ± 5.62° vs. 16.42 ± 4.49°, *p* = 0.000) (Table 4), which manifested tooth protrusion and proclination. Additionally, Table 2 shows that the mean APT are significantly smaller in Class III canine relationship (Class I: 8.47 ± 1.93 mm vs. Class III: 6.79 ± 1.65 mm, *p* = 0.000), and the proportion exhibiting thinner ABT and thicker APT was significantly larger in Class III canine relationship. Moreover, SA exhibited a strong positive correlation with the APT and a moderate negative correlation with the ABT (R = 0.723 and − 0.554) regardless of canine relationships (Table 6). In other words, at the apex, the more labially inclined the maxillary central incisor is, the thinner the palatal wall and the thicker the buccal wall will be. Therefore, for labially inclined maxillary frontal teeth, especially in patients with Class III canine relationship or any Class III occlusal traits, thinner alveolar palatal bone walls can possibly be anticipated.

**Table 6:** Correlations between bone thickness and the sagittal angulation of upper central incisor using Pearson correlation test

	Correlation coefficient R	*p*
MBT versus SA	− 0.153	0.115
MPT versus SA	0.433	0.000*
ABT versus SA	− 0.554	0.000*
APT versus SA	0.723	0.000*

*MBT* mid-root buccal thickness, *SA* sagittal angulation, *MPT* mid-root palatal thickness, *ABT* apical buccal thickness, *APT* apical palatal thickness

*Statistically significant (*p* < 0.05)

From the restoration perspective, it is always better to place implants into the exact extraction socket at the same angulation so that the screw access can emerge at the crown’s cingulum. According to Wang et al. [18], when the angle between the long axes of tooth and alveolar bone was < 10 degrees, it would be relatively easy to insert implants into extraction socket in the same direction as the root but slightly palatally. However, more than 80% of maxillary anterior teeth were positioned against the labial cortical plate (81.1%) [24] or buccally in the osseous housing (94%) [25]. The current study also showed that less than half of the study samples had facial bone wall > 1 mm. Therefore, a palatally positioned osteotomy is suggested to keep a safe distance from the buccal plate. The relatively abundant palatal bone helps implant engage more native bone, which improves the primary stability and avoids the pressure exerted by implants on the buccal plate [26].

According to this study, the upper central incisors in Class I canine relationship are relatively more lingually inclined and have a thicker alveolar palatal bone at both mid-root and apex level compared to Class III canine relationship. Notably, 5.6% of the cases with Class I canine relationship had SA larger than 25 degrees (Table 5). Thus, palatal insertion of implants for Class I population is feasible, and the angle between the long axes of the tooth and implant can be corrected by an angled abutment and cemented crown. In contrast, patients with Class III canine relationship usually manifest more protrusive upper frontal teeth, which indicates thinner palatal bone according to our study. If the same palatal engagement rule is applied, palatal perforation may occur during immediate implant surgical procedures unless a narrow-diameter implant is used. For cases with severe skeletal Class III malocclusion, a delayed approach would be recommended after orthodontic and orthognathic therapy.

The size and shape of the palate are closely related to craniofacial morphology [27]. Studies have shown that the thickness of the palatine process is influenced by vertical or sagittal skeletal configurations. Patients with skeletal class III malocclusion are likely to have mandibular prognathism [28] or maxillary hypoplasia [29] which may be accompanied by palatal hypoplasia [27]. Palatal bone thickness was found thinner in Class I malocclusion with open vertical skeletal configuration (*p* < 0.05) [30]. In hyperdivergent women, available palatal bone may be smaller than normal in the middle and posterior areas [31]. Moreover, the alveolar bone thickness around incisors can also be originally or developmentally thinner in skeletal class III malocclusion patients [32]. In this study, maxillary central incisor’ palatal alveolar bone with Class III canine relationship were significantly thinner at both mid-root and apical level, which may be accounted for by the palatal hypoplasia associated with Class III skeletal deformity as described in previous studies.

There are some limitations to the present study. First, the current results were based on visual measurements, the relatively low resolution and pixel size of 0.300 mm of CBCT interfered with the observer’s judgements. Second, the study did not include samples with distal (Class II) canine relationship which can be seen in both Angle Class II, division 1 and 2 malocclusions. Therefore, further studies with larger sample sizes and a more precise classification are required.

## Conclusions

Canine relationship can reflect different craniofacial growth patterns and dentoalveolar characteristics of maxillary incisors like tooth inclination and alveolar bone volume. Maxillary central incisors of patients with Class III canine relationship were found to have thinner palatal bone plate and were significantly more labially inclined compared to Class I canine relationship. Besides, the sagittal angulation (SA) of maxillary incisors mainly influences the buccal and palatal alveolar bone width at apex (ABT and APT). A smaller SA is correlated with a thicker buccal bone plate at apex and a thinner palatal bone plate. Clinicians should avoid palatal perforation during immediate implantation at sites of originally protrusive maxillary incisors.

## Data Availability

The datasets used and/or analysed during the current study can be later deposited publicly and available from the corresponding author on reasonable request.

## Abbreviations

CBCT: Cone-beam computed tomography.
DICOM: Digital imaging and communications in medicine
MBT: Mid-root buccal thickness.
MPT: Mid-root palatal thickness.
ABT: Apical buccal thickness.
APT: Apical palatal thickness.
SA: Sagittal angulation.
